# Traditional Chinese Medicine in Treating Primary Podocytosis: From Fundamental Science to Clinical Research

**DOI:** 10.3389/fphar.2022.932739

**Published:** 2022-08-08

**Authors:** Lirong Lin, En Tian, Jiangwen Ren, Zhifeng Wu, Junhui Deng, Jurong Yang

**Affiliations:** ^1^ Department of Nephrology, The Third Affiliated Hospital of Chongqing Medical University (General Hospital), Chongqing, China; ^2^ Department of Nephrology, Rheumatism and Immunology, Jiulongpo District People’s Hospital of Chongqing, Chongqing, China

**Keywords:** traditional Chinese medicine, minimal change disease, idiopathic membranous nephropathy, focal segmental glomerulosclerosis, podocyte

## Abstract

Podocytes form a key component of the glomerular filtration barrier. Damage to podocytes is referred to as “podocyte disease.” There are many causes of podocyte injury, including primary injury, secondary injury, and gene mutations. Primary podocytosis mostly manifests as nephrotic syndrome. At present, first-line treatment is based on glucocorticoid administration combined with immunosuppressive therapy, but some patients still progress to end-stage renal disease. In Asia, especially in China, traditional Chinese medicine (TCM) still plays an important role in the treatment of kidney diseases. This study summarizes the potential mechanism of TCM and its active components in protecting podocytes, such as repairing podocyte injury, inhibiting podocyte proliferation, reducing podocyte apoptosis and excretion, maintaining podocyte skeleton structure, and upregulating podocyte-related protein expression. At the same time, the clinical efficacy of TCM in the treatment of primary podocytosis (including idiopathic membranous nephropathy, minimal change disease, and focal segmental glomerulosclerosis) is summarized to support the development of new treatment strategies for primary podocytosis.

## Introduction

Podocytes, i.e., glomerular visceral epithelial cells, adhere to the surface of the glomerular basement membrane (GBM). They are named after their “pseudopodia-like” protrusions in the cytoplasm and are multipotent cells with a complex terminal differentiation structure (Torban et al., 2019). Podocytes form an important part of the glomerular filtration barrier, together with the GBM and endothelial cells. The main functions of podocytes include the following: 1) protein filtration by forming a molecular weight barrier and a charge barrier; 2) changing the ultra-filtration coefficient and regulating glomerular filtration; 3) resisting the pressure in the glomerulus and maintaining the spatial structure of the glomerular capillary loop; 4) secreting structural components and degrading enzymes of the GBM, thereby participating in the maintenance of the metabolic balance; and 5) synthesizing and secreting vascular endothelial growth factor to maintain the functional integrity of glomerular endothelial cells (Garg, 2018; Sun et al., 2021).

Podocyte injury is the main mechanism of glomerular proteinuria, which is closely related to the occurrence and development of a variety of glomerular diseases. In 2002, the glomerular disease characterized by the change in podocyte structure and function was named “podocytosis” for the first time. It is characterized by a reduction in podocyte number or density, thickening of the GBM, a change in glomerular matrix composition, and foot process fusion (Lu et al., 2019; Sun et al., 2021). When podocytes are injured, foot process fusion, apoptosis, developmental arrest, epithelial–mesenchymal transition (EMT), idiopathic membranous nephropathy (IMN), minimal change disease (MCD), focal segmental glomerulosclerosis (FSGS), and collapsing glomerulopathy (CG) can occur with the abovementioned pathological changes in podocytes, which are characteristic of podocyte disease (Barisoni et al., 2009; Lu et al., 2019).

## Factors of Podocyte Injury

Podocyte injury can be caused by a variety of etiologies. The most common is immune injury, including immune complex deposition and *in situ* immune complex formation. Other factors, such as drugs, other toxic agents, infections, gene mutations, hemodynamic factors, and protein overload, can cause glucose and lipid metabolism disorders (Lin et al., 2021), which lead to podocyte hypertrophy (Zhou et al., 2021), podocyte foot process disappearance (Tojo, 2019; Purohit et al., 2021), EMT (Ying and Wu, 2017), and podocyte abscission and apoptosis (Dai et al., 2017; Gil et al., 2021). The causes of common podocyte diseases, such as immune damage, drugs, toxic agents, and infections, are summarized below.

### Immunologic Injury

In many immune-mediated glomerulonephritides, podocytes are important targets of immune damage (Salvadori and Tsalouchos, 2021). In the early stage of injury, there are some structural changes in podocytes, including rearrangement of the actin cytoskeleton and loss of slit diaphragm integrity, which prevent podocyte detachment from the GBM but may cause podocyte foot process effacement. Foot processes disappear over time (Kriz et al., 2013; Kriz and Lemley, 2017). As the injury continues or worsens, however, podocytes will eventually separate, leading to glomerular segmental sclerosis or global sclerosis (Nagata, 2016).

Membranous nephropathy is a typical glomerular disease caused by immune complex deposition. The root cause of proteinuria in idiopathic membranous nephropathy (IMN) is the targeted binding of autoantibodies to podocytes (Nieto-Gañán et al., 2022). At present, known podocyte autoantigens include antigen-neutral endopeptidase (NEP) (Debiec et al., 2002); M-type receptor for secreted phospholipase A2 (PLA2R1) (Beck et al., 2009); thrombospondin type-1 domain-containing 7A (THSD7A) (Tomas et al., 2014); and other MN-associated antibody-targeting podocyte antigens, such as neural epidermal growth factor-like 1 protein (NELL-1) (Sethi et al., 2020), exostosin 1 and exostosin 2 (Ronco et al., 2006), neural cell adhesion molecule 1 (NCAM-1) (Vivarelli et al., 2015), and contactin 1 (CNTN1) (Nazarali et al., 2020). NEP is an antigen found in the neonatal kidney (Debiec et al., 2002), which participates in the catabolism of vasoactive regulatory peptides and blocks peptide signal transduction on the cell surface (Ronco et al., 2006). When the NEP antibody binds to podocytes, kidney damage may occur because the enzyme activity affecting glomerular hemodynamics is blocked (Debiec et al., 2002), which is associated with the enzyme activity that maintains endothelial permeability (Vivarelli et al., 2015), finally leading to MN (Ronco et al., 2006). PLA2R1 and THSD7A play an important role in maintaining the normal arrangement of podocytes, the slit diaphragm, and adhesion between podocytes and the GBM. THSD7A is expressed in human and mouse podocytes, enhances podocyte adhesion, promotes the surface adhesion of other type IV collagen coatings, reduces the migration ability, and plays an important role in podocyte cytoskeleton regulation (Tomas et al., 2014; Tomas et al., 2016; Herwig et al., 2019). Anti-PLA2R1/THSD7A antibodies bind to corresponding antigens of podocytes to form immune deposits, resulting in impaired filtration membrane integrity, thickening of the GBM, and changes in podocyte morphology and function, such as increased podocyte volume, loss of foot processes, and massive proteinuria (Fresquet et al., 2017; Liu et al., 2020; Meyer-Schwesinger et al., 2020). NELL-1 is a secreted protein (Li et al., 2019), which exists in the blood and kidneys of MN patients (Zhang et al., 2010). Most NELL-1-positive MN patients do not have autoimmune diseases, but NELL-1 positivity is associated with a higher incidence of tumors (Li et al., 2019; Bobart et al., 2021). As NELL-1 is the target antigen of PMN, these results need to be verified by studies that include larger samples. It has been hypothesized that NELL-1 forms an immune complex that is detached from podocytes and deposited in the GBM, causing proteinuria (Gu et al., 2021). It was found that most exostosin-positive patients are complicated by systemic lupus erythematosus or other autoimmune diseases, so NELL-1 might be a target antigen of secondary membranous nephropathy (Ronco et al., 2006), but the mechanisms underlying podocyte injury are not clear. NCAM-1 expression was found in the renal tissues of patients with membranous lupus nephritis and a few primary membranous nephropathies (PMNs), and the antibody was mainly immunoglobulin G1 (Caza et al., 2021; Wu et al., 2021). NCAM-1-related MN mainly manifests as GBM thickening and mesangial proliferation (Sethi, 2021). CNTN1 is a common target antigen with peripheral nerves and podocytes of patients with neurological diseases and MN, and the antibody is mainly immunoglobulin G4 (Le Quintrec et al., 2021). CNTN1 disrupts the adhesion function and normal structure of podocytes by affecting the Notch1 signaling pathway (Debiec and Ronco, 2021). It is very difficult to strictly distinguish between PMN and secondary membranous nephropathy using these target antigens alone. In the future, it may be useful to use a combination of target antigens to distinguish between the types of MNs.

### Drugs and Toxic Agents

Drugs that include interferon (IFN), non-steroidal anti-inflammatory drugs (NSAIDs) (indomethacin and celecoxib), antiviral drugs, mammalian target of rapamycin (mTOR) inhibitors (sirolimus), calcineurin inhibitors (CNIs), androgenic anabolic steroids, antineoplastic drugs (pazopanib), and pamidronate may have an effect on glomerular podocytes. Some toxic agents, such as lithium and organic mercurials, have also been reported to damage podocytes. The following is a brief description of the causes of drug-induced renal podocytosis.

A study reported that 11 patients who received IFN treatment developed proteinuria 4–12 months after treatment. Renal histopathological examinations revealed FSGS, and renal ultrastructural pathological examinations also showed that the foot process was lost, accompanied by endothelial tubuloreticular inclusions. After stopping IFN treatment, about 70% of patients with renal disease were relieved. At the same time, 21 patients with previously reported renal disease caused by IFN were reviewed, including 8 cases of MCD, 1 case complicated by acute interstitial nephritis, and 12 cases of FSGS. A total of 11 patients received prednisone treatment, and 45% of these patients (5/11) achieved remission (Markowitz et al., 2010). The mechanisms underlying IFN-induced damage to podocytes may include increasing the permeability of podocytes, inducing podocyte death, interfering with the differentiation of renal progenitor cells into mature podocytes (Migliorini et al., 2013), stimulating the podocyte secretion process (INF-γ) (Mühlig et al., 2020), and inhibiting autophagy in human podocytes by producing reactive oxygen species (IFN-α) (Zhou et al., 2019).

Kidney damage by NSAIDs has attracted significant attention. NSAIDs can not only lead to acute tubulointerstitial kidney injury (Dreischulte et al., 2015; Zhang et al., 2017; Yu et al., 2020) but they also cause MCD, and their long-term use can lead to chronic kidney disease (CKD) (Hörl, 2010; Chiu et al., 2015). The probability of developing CKD with NSAID use varies according to the type of drug used and the patient’s basic renal function (Gicchino et al., 2021). An observational study of preterm infants treated with indomethacin found significantly greater podocyte and urinary protein excretion in treated infants than in other infants (Kent et al., 2012). In addition to hemodynamic changes (Drożdżal et al., 2021), the most important cause of NSAID-induced CKD is damage to glomerular podocytes and the GBM. After 20 weeks of celecoxib treatment in diabetic rats, the number of glomerular podocytes decreased, the GBM was thinner and its diameter was decreased, and the mesangial matrix was proliferated (Nasrallah et al., 2013). Therefore, the decrease in glomerular podocytes and the structural changes that occur with NSAID treatment may cause kidney disease.

Pathological changes in MN (Murakami et al., 2018), FSGS (Hogan et al., 2017), and lupus-like nephritis (Sise et al., 2016) occurred in patients with chronic hepatitis C infection after receiving direct-acting antivirals (including ribavirin, simeprevir, sofosbuvir, and ledipasvir). Kidney damage from such drugs may include direct podocyte toxicity and immune complex deposition. Inhibition of mTOR activation reduces proteinuria and glomerulosclerosis (Miesen et al., 2020), but complete reduction in mTOR activity may lead to glomerular disease progression (Letavernier et al., 2007; Cai et al., 2011), indicating that the therapeutic window of the drug is narrow. After renal transplantation, patients treated with mTOR inhibitors have increased urinary protein excretion, podocyte damage, and FSGS changes (Letavernier and Legendre, 2008). A study found that, after renal transplantation, the number of terminal deoxynucleotidyl transferase-mediated dUTP nick-end labeling-positive cells was significantly greater in patients treated with sirolimus compared to the control group (Munivenkatappa et al., 2010), suggesting that the apoptosis of renal tubular epithelial cells and podocytes was enhanced. In addition, mTOR inhibitors induce proteinuria by reducing the expression of podocyte hiatal membrane-related molecules (Kim et al., 2017), which induces proteinuria in a dose-dependent manner (Stallone et al., 2011). The non-immune effects of CNIs are nephrotoxic, including tubular atrophy, interstitial inflammation, podocyte damage, and hyaline hyperplasia of arterioles (Gooch et al., 2017). A study found that cyclosporine A-induced expression of CD44 in mice increased with increasing doses, and the foot process, balloon adhesion, and segmental sclerosis in the glomerulus disappeared (Hayashi et al., 2019).

After long-term abuse of anabolic steroids, a patient developed nephrotic syndrome. Renal biopsy revealed glomerulomegaly and FSGS (Herlitz et al., 2010; Pendergraft et al., 2014). This may be related to the alteration of glomerular hemodynamics caused by weight gain after hormone application and the decrease in proteinuria after hormone withdrawal (Harrington et al., 2011). It has been reported that, following the application of anti-tumor drugs (pazopanib), patients may develop nephrotic syndrome. The pathological manifestations are the disappearance of the foot process, GBM double-track syndrome, and thrombotic microvascular disease, and proteinuria gradually decreases after drug discontinuation (Maruyama et al., 2018). It has been reported that collapse-type FSGS can appear after high-dose pamidronate treatment (Markowitz et al., 2001; Barri et al., 2004; Dijkman et al., 2006), but the specific mechanism of podocyte injury has not been studied.

Short-term and low-dose lithium preparations can resist proteinuria by targeting glycogen synthase kinase 3β, promoting the repair of kidneys and podocytes, and protecting the kidneys (Xu et al., 2014; Bao et al., 2015; Guo et al., 2016). However, long-term lithium exposure may lead to the disappearance of the glomerular podocyte foot process and MCD (Tandon et al., 2015; Gong et al., 2016; Bgatova and Taskaeva, 2020). Mercurials in cosmetics may also lead to MN.

### Infectious Diseases

Viral infections, including human immunodeficiency virus (HIV), severe acute respiratory syndrome coronavirus 2 (SARS-CoV-2), cytomegalovirus (CMV), and parvovirus B19, are the most likely cause of glomerular podocyte damage and collapsing FSGS (Muehlig et al., 2022). The main pathological features of HIV-associated nephropathy are the disappearance of the podocyte foot process, podocyte dedifferentiation, proliferation, apoptosis, podocyte detachment, and massive proteinuria (Canaud et al., 2014). The mechanism of podocytosis mainly includes the following aspects: 1) direct infection of podocytes and destruction of the cytoskeleton of podocytes, resulting in damage to the glomerular filtration barrier (Ospina Stella and Turville, 2018); 2) podocyte injury and dysfunction caused by the HIV-related proteins, including Nef and Vpr (Gu et al., 2013; Tan et al., 2013; Gbadegesin et al., 2014; Hall et al., 2018a; Rednor and Ross, 2018); and 3) podocyte proliferation and dedifferentiation caused by HIV-induced vascular endothelial growth factor (Veron et al., 2010; Wang et al., 2015a).

In addition to acute tubulointerstitial nephritis (Cheng et al., 2020; Lin et al., 2020), the renal damage caused by SARS-CoV-2 is also very apparent; the main pathological change is collapsing FSGS (Magoon et al., 2020; Charytan et al., 2021; Shetty et al., 2021). Virus particles were found in kidney cells by electron microscopy, and viral proteins were detected by immunohistochemistry, confirming that SARS-CoV-2 can directly infect the kidneys (Khan et al., 2020; Hassler et al., 2021). Podocytes of patients with coronavirus disease 2019 exhibit high expression of angiotensin-converting enzyme 2, cellular transmembrane serine proteases, and furin, which are related to the invasion of SARS-CoV-2 into podocytes (Sallenave and Guillot, 2020; Stasi et al., 2020). Collapsing kidney disease caused by SARS-CoV-2 is often accompanied by mutations in the apolipoprotein L1 (*APOL1*) gene, which may lead to mitochondrial dysfunction and reduced cell membrane integrity through various mechanisms, thus resulting in podocyte destruction (Kudose et al., 2020; Friedman and Pollak, 2021).

Glomerular diseases can also be caused by other viral infections (Chandra and Kopp, 2013), such as parvovirus B19 mesangial proliferative glomerulonephritis, which is mainly characterized by IgA deposition. CMV infection is characterized by focal segmental mesangial hyperplasia with sclerosis, disappearance of foot processes, and membranous nephropathy. Thrombotic microangiopathy is the main pathological change (Jacob et al., 2020). Epstein–Barr virus (EBV) infection is mainly characterized by membranous nephropathy and crescentic glomerulonephritis, and dengue virus infection is mainly characterized by FSGS (Araújo et al., 2018). A study found that Zika virus can directly infect podocytes to cause podocyte disease (Alcendor, 2017). Respiratory syncytial virus infection can cause podocyte foot process disappearance and mesangial matrix hyperplasia-like pathological changes (Zhai et al., 2016). Infection by various viruses in children can cause MCD or FSGS, including HIV, hepatitis B virus, hepatitis C virus, parvovirus B19, CMV, and EBV (Dettmar and Oh, 2016).

A study found that *APOL1* is the most common susceptibility gene for podocytosis (Friedman and Pollak, 2021; Watanabe et al., 2021), which is closely related to collapsing FSGS, hypertensive renal injury, and HIV-related kidney diseases (Kopp et al., 2011; Lipkowitz et al., 2013; Kasembeli et al., 2015; Reidy et al., 2018). With the widespread application of whole-gene detection technology, gene mutations related to podocytosis have been gradually discovered and recognized. At present, > 50 gene mutations related to podocytes have been found in children and adults’ hormone-resistant nephrotic syndrome (Chen and Liapis, 2015). The pathogenic genes mainly encode pore membrane proteins (NPHS1, NPHS2, CD2AP, and PTPRO) (Delville et al., 2014; Liu et al., 2021a; Maier et al., 2021), signal transduction-related proteins (TRPC6) (Hall et al., 2019), cytoskeletal proteins (PODXL, ACTN4, INF2, MYH9, and ANLN) (Hall et al., 2018b; Cechova et al., 2018; Shao et al., 2019; Refaeli et al., 2020; Subramanian et al., 2020), transcription factor-related proteins (WT1 and PAX2) (Niaudet and Gubler, 2006; Hu et al., 2021), and cellular/extracellular matrix-related, mitochondrial, and lysosomal proteins (Chen and Liapis, 2015).

## Protective Effects of Traditional Chinese Medicine and Active Ingredients on Podocyte Injury

The prognosis of MCD treated with glucocorticoids (GCs) is relatively good, and disease progression is slow (Vivarelli et al., 2017). The prognosis of primary FSGS varies according to its response to GCs (Mason et al., 2020; Zhao and Liu, 2020). Steroid-resistant FSGS is an independent risk factor for decreased renal function (Rosenberg and Kopp, 2017; Ying et al., 2021). At present, the main methods for the treatment of MCD, FSGS, and MN include angiotensin-converting enzyme inhibitors or angiotensin receptor blockers, steroid hormones, CNIs (cyclosporine or tacrolimus), cyclophosphamide, or CD20 monoclonal antibody. However, GCs and immunosuppressants also have side effects, such as severe infection and disorders of glucose and lipid metabolism. TCM has made certain achievements in the fundamental research and clinical treatment of primary podocytosis (Kidney Disease: Improving Global Outcomes (KDIGO) Glomerular Diseases Work Group, 2021). Below, we discuss 1) the research progress of TCM and its active ingredients in the prevention and treatment of podocyte injury and 2) the analysis of its efficacy in primary podocyte disease to support the development of new treatment strategies for podocytosis.

### Podocyte Injury Repair

Tritolide can significantly reduce proteinuria, reduce glomerular hypertrophy, and repair podocyte damage in db/db mice (Gao et al., 2010). Qi Dan Fang is composed of *Astragalus* and *Salvia miltiorrhiza* at a ratio of 5:1, which can upregulate the protein expression of Bcl-2 in rats with adriamycin nephrotic syndrome, reduce podocyte damage, and restore glomerular filtration function (Wu et al., 2014). Tongxinluo inhibits inflammatory cell infiltration in hypertensive kidney damage in spontaneously hypertensive rats; reduces the activity of oxidative stress damage markers; and decreases the expression of α-smooth muscle actin (α-SMA), extracellular matrix protein, transforming growth factor β1, and other chemical markers to reduce podocyte injury (Luo et al., 2015). The active ingredient of ginseng, ginsenoside Rg1, exerts protective effects on podocytes by reducing the oxidative stress response of D-galactose-induced subacute mouse kidney injury (Fan et al., 2016). Sirtuin-1 deacetylase plays a key role in renal cell protection mainly by reducing cellular stress responses. Resveratrol and puerarin are known sirtuin-1 agonists that repair podocyte damage in type I diabetic mice (Zhong et al., 2018). Berberine promotes mitochondrial energy homeostasis and fatty acid oxidation by ameliorating mitochondrial dysfunction and fatty acid oxidation defects, thereby promoting post-injury podocyte repair in db/db mice (Qin et al., 2020). Yiqi Huoxue Decoction consists of >10 kinds of herbal medicines, and luteolin, wogonin, formononetin, and verbasin were found to be the top potential active compounds by ultra-high-performance liquid chromatography–mass spectrometry. Yiqi Huoxue Decoction attenuates and repairs podocyte injury through the phosphatidylinositol 3-kinase–RAC serine/threonine protein kinase (PI3K/AKT) and nuclear factor kappa-B (NF-κB) signaling pathways (Feng et al., 2022).

### Podocyte Apoptosis and Excretion Reduction

Astragaloside IV is an active ingredient of *Astragalus membranaceus*, which inhibits the expression of glucose-regulated protein 78 and oxygen-regulated protein 150 by inhibiting the expression and phosphorylation of PERK and JNK, inhibiting the apoptosis of podocytes, and reducing podocyte excretion in streptozotocin-induced diabetes nephropathy in rats (Wang et al., 2015b). *Tripterygium wilfordii* and its active components upregulate autophagy in diabetic kidney disease (DKD) mice through the mTOR/Twist1 signaling pathway, reduce podocyte transdifferentiation and apoptosis (Tao et al., 2021), reduce interleukin-4 overexpression, enhance cell viability, and inhibit podocyte apoptosis (Li et al., 2020). Salvianolic acid A is the main active component of *Salvia miltiorrhiza*. By inhibiting the expression of soluble urokinase-type plasminogen activator receptor, it increases the expression of the podocyte-related protein nephrin, reduces podocyte apoptosis, and promotes podocyte repair (Li et al., 2021a). Ginsenoside Rb1 can reduce podocyte excretion by reducing apoptosis and mitochondrial damage induced by high glucose levels in diabetic nephropathy (He et al., 2022). *Abelmoschus manihot* flowers can improve podocyte apoptosis by reducing inflammation and oxidative stress (Li et al., 2021b).

Modified Huangqi Chifeng Decoction improves proteinuria and reduces podocyte excretion by inhibiting excessive autophagy in doxorubicin-induced nephropathy in rats through the PI3K/mTOR signaling pathway (Yu et al., 2018). Yu Nu regulates autophagy stabilization, inhibits apoptosis, and reduces podocyte excretion in Goto–Kakizaki rats by reducing mTOR levels (He et al., 2020). *Paecilomyces cicadae*-fermented Radix Astragali can enhance the autophagy of podocytes in diabetic mice by inhibiting the PI3K/AKT/mTOR signaling pathway, thereby reducing podocyte apoptosis and improving the renal structure of diabetes nephropathy (Yang et al., 2020). Mahuang Fuzi and Shenzhuo Decoction can inhibit Wnt/β-catenin signal activation of podocytes in mice stimulated by high glucose, enhance podocyte autophagy, and reduce podocyte damage (Dai et al., 2020). Yiqi Jiedu Huayu Decoction can promote autophagy in streptozotocin (STZ)-induced diabetes mellitus rats by upregulating the expression of autophagy-related proteins, regulating the activity of the PI3K/Akt and AMPK pathways, inhibiting the mTOR pathway, and promoting autophagy of STZ-induced diabetic nephropathy (Xuan et al., 2021).

### Upregulation of Podocyte-Associated Proteins

Podocyte-associated proteins play an important role in maintaining glomerular filtration function. Among them, pore membrane-related protein molecules, especially nephrin, podocin, and CD2AP, constitute a “zipper” structure, which is important in maintaining pore membrane integrity. It was found that Hyperoside (Zhang et al., 2016), Gushen Jiedu Capsule (Zhang et al., 2020), Wenshen Jianpi Recipe (Cao et al., 2019), Qi Dan Fang (Wu et al., 2014), Genipin (Qiu et al., 2012), Zhen-wu-tang (Cai et al., 2010), and other preparations can increase the messenger RNA expression of cleft septum proteins (including nephrin, podocin, CD2AP, and WT1) in diabetic mice to improve podocyte injury and protect kidneys.

Huangqihuang protects podocytes by inhibiting the p-ERK/CHOP signaling pathway and upregulating the expression levels of podocin, nephrin, and synaptopodin in a tunicamycin-induced rat model (Li et al., 2016). Jiedu Tongluo Baoshen (JDTB) contains 77 active ingredients to promote the expression of the podocyte-related proteins, podocin, nephrin, and WT-1, so as to reduce podocyte injury. Meanwhile, JDTB activates autophagy-related proteins, such as beclin-1, LC3, and p62, to enhance podocyte autophagy and reduce podocyte injury (Jin et al., 2022). Atractylodis Rhizoma water extract upregulates podocin, nephrin, and CD2AP and promotes apoptosis in fructose-fed rats by inhibiting TRPC6/p-CAMK4 signal transduction. α-Actinin-4 and other podocyte proteins are expressed to prevent glomerular podocyte injury (Chen et al., 2021). Bu-Shen-Huo-Xue Decoction upregulates the podocyte markers nephrin and podocin in high-fat diet/STZ-induced diabetic mice and downregulates mesenchymal markers. α-SMA and fibroblast-specific protein 1 are expressed to improve renal function and inhibit podocyte EMT (Wang et al., 2020). Rhodiola can increase the expression levels of nephrin and podocin and protect podocytes by regulating SIRT1/PGC-1α-mediated mitochondrial bioactivity and inhibiting the β-catenin signaling pathway (Huang et al., 2019; Xue et al., 2019).

### Skeleton Maintenance of Podocytes

The podocyte cytoskeleton plays an important role in maintaining the normal morphology of podocytes. Mature podocytes express vimentin and desmin in the cell body and foot process. TCMs repair the podocyte skeleton by inhibiting renin–angiotensin–aldosterone system activation, cytokine overexpression, and other mechanisms so as to improve podocyte injury (Dou et al., 2009). Studies have found that Dahuang Zhechong Pill can significantly reduce the expression of desmin in a rat nephrotic model with adriamycin, maintain podocyte function, and reduce urinary protein excretion (Chen et al., 2008). Wulingsan (Gorei-San) regulates renal nephrin gene expression in rats with doxorubicin-induced nephrotic syndrome by inhibiting the overactivity of the renin–angiotensin system, maintaining the normal cytoskeleton of podocytes, and ameliorating doxorubicin-induced podocyte injury (He et al., 2008). Huaiqihuang Granule upregulates the expression of nephrin and podocin in rats with adriamycin nephropathy and maintains the integrity of podocyte synovium and cytoskeleton (Sun et al., 2011). Huangqi Guizhi Wuwu Decoction upregulates the expression of the cytoskeletal proteins nephrin, podocin, and ACTN4 in podocytes through the AT1R–nephrin–c-Abl pathway and downregulates the expression levels of TNF-α, AT1R, and c-Abl to alleviate the damage of cytoskeletal proteins in podocytes (Liu et al., 2021b).

### Inhibition of Podocyte Proliferation

Glomerular podocytes are highly differentiated terminal cells. Under pathological conditions, abnormal proliferation and cell phenotypic changes can occur due to podocyte dysfunction. Some TCMs can participate in protein synthesis, energy metabolism, and cell cycle regulation of podocytes and mesangial cells through the Pik/Akt/mTOR signaling pathway so as to inhibit the excessive proliferation of podocytes (Yang et al., 2015). Dipeptidyl peptidase 4 (DDP4) is specifically upregulated in DKD podocytes, which may be related to podocyte proliferation. Through analysis of the TCM Systematic Pharmacology database, it was found that quercetin, methyl rosmarinate, kaempferol, diosgenin, and *Acacia* may inhibit DPP4 activity and the proliferation of DKD podocytes (Wang et al., 2021). High-dose *Tripterygium wilfordii* can improve the overexpression of nephrin in adriamycin-treated rats and inhibit abnormal proliferation of podocytes so as to reduce proteinuria and glomerulosclerosis (Wan et al., 2014). Yuxue Decoction can significantly inhibit the lipid-induced proliferation of mouse podocytes (Chen et al., 2007). The protective mechanism of TCM on podocyte injury is shown in Table 1.

**TABLE 1:** Protective mechanism of TCM on podocyte injury.

Name	Compound TCM prescriptions	Main active ingredients	Protective mechanism of podocytes	References
Wulingsan (Gorei-San)	Alismatis Rhizome, Poria, Polyporus, Atractylodis Rhizome, Cinnamomi Ramus	—	Skeleton maintenance of podocytes	He et al. (2008)
Tripterygium wilfordii Hook F		Triptolide	Podocyte injury repair, podocyte apoptosis, and excretion reduction	Gao et al. (2010) Li et al. (2020)
Genipin	—	Geniposide	Upregulation of podocyte-associated proteins	Qiu et al. (2012)
*Astragalus* membranaceus (Fisch) Bge	—	Astragaloside IV	Podocyte apoptosis and excretion reduction	Wang et al. (2015)
Tongxinluo	—	—		Luo et al. (2015)
Ginseng	—	Ginsenoside Rg1	Podocyte injury repair, podocyte apoptosis, and excretion reduction	Fan et al. (2016) He et al. (2022)
Huaiqihuang	Tree ear fungus, medlar, and Huang Jing	—	Upregulation of podocyte-associated proteins	Li et al. (2016)
Modified Huangqi Chifeng decoction	*Astragalus* membranaceus Bge, Euryale ferox Salish, Rosae Laevigatae Michx, Paeonia lactiflora Pall, Saposhnikovia divaricata Schischk, Pheretima vulgaris Chen, and Hedyotis diffusa Wild	—	Podocyte apoptosis and excretion reduction	Yu et al. (2018)
Salidroside	—	—	Upregulation of podocyte-associated proteins	Xue et al. (2019), Huang et al. (2019)
Wenshen Jianpi recipe	Aconiti Lateralis Radix Praeparata, Zingiber officinale Roscoe, Radix Codonopsis, Rhizoma Atractylodis macrocephalae, Poria cocos, Radix Paeoniae Alba, *Glycyrrhiza* uralensis	—	Upregulation of podocyte-associated proteins	Cao et al. (2019)
Berberine	—	—	Podocyte injury repair	Qin et al. (2020)
Gushen Jiedu capsule	Semen Euryales, Fructus Rosa laevigata, Rhizome Coptis chinensis, Rhizome Rheum tanguticum, Radix *Astragalus* membranaceus, and Radix Angelica sinensis	—	Upregulation of podocyte-associated proteins	Zhang et al. (2020)
Yu Nu Compound	—	—	Podocyte apoptosis and excretion reduction	He et al. (2020)
Abelmoschus manihot flowers	—	—	Podocyte apoptosis and excretion reduction	Li et al. (2021)
Paecilomyces cicadae-fermented Radix astragali	—	—	Podocyte apoptosis and excretion reduction	Yang et al. (2020)
Mahuang Fuzi and Shenzhuo Decoction	—	—	Podocyte apoptosis and excretion reduction	Dai et al. (2020)
Bu-Shen-Huo-Xue Decoction	Psoraleae Fructus, Eucommia Cortex, Lycii Fructus, Cistanches Herba, Rehmanniae Radix Praeparata, Cuscutae Semen, and Corni Fructus, comprising Angelica Sinensis Radix, Angelicae Pubescentis Radix, Carthami Flos, and Myrrh	—	Upregulation of podocyte-associated proteins	Wang et al. (2020)
Yiqi Jiedu Huayu Decoction	Pueraria *montana* var. lobata (willd.) Maesen and S. M. Almeida ex Sanjappa and Predeep, Coptis chinensis Franch, and Morus alba L	—	Upregulation of podocyte-associated proteins	Xuan et al. (2021)
Atractylodis rhizoma water extract	—	—	Upregulation of podocyte-associated proteins	Chen et al. (2021)
Huangqi Guizhi Wuwu Decoction	Radix astragali, Ramulus cinnamomi, Radix paeoniae alba, Rhizoma zingiberis recens, and Fructus jujubae	—	Skeleton maintenance of podocytes	Liu et al. (2021)
Lobeliae	—	—	Inhibition of podocyte proliferation	Wang et al. (2021)
Jiedu Tongluo Baoshen formula	—	—	Upregulation of podocyte-associated proteins	Jin et al. (2022)
Hyperoside	—	Active flavonoid glycoside	Upregulation of podocyte-associated proteins	Zhang et al. (2016)

## Clinical Studies on Treating Podocytopathies With TCM

IMN, MCD, and FSGS are characterized by a decrease in the number or density of podocytes, thickening of the GBM, changes in the composition of the glomerular matrix, and fusion of the foot processes, which are typical of podocytosis. The main clinical feature is nephrotic syndrome. According to the guidelines, the main treatment for podocyte disease is GC combined with immunosuppressive agents. However, there are many complications and poor curative effect in some patients. With the understanding and research of TCM, the efficacy of traditional Chinese medicine in podocytosis has been recognized.

### Idiopathic Membranous Nephropathy

TCMs can improve kidney function by regulating the body’s immunity and reducing the inflammatory response. Studies have shown that TCM can be used to effectively treat IMN (Feng et al., 2020). A meta-analysis including 29 studies showed that the total effective rate and cure rate of IMN patients were significantly increased after combining TCM and immunosuppressive therapy, and combined therapy also significantly reduced the recurrence rate and adverse events. However, due to the small sample size of all studies included in this meta-analysis, and the lack of long-term follow-up and scientific design of the research results, the reliability of the research results is affected (Lu et al., 2021).

The treatment scheme of supplementing Qi and activating blood circulation by using *Astragalus membranaceus*, *Angelica sinensis*, *Ligusticum chuanxiong*, *Codonopsis pilosula, Atractylodes macrocephala*, and *Salvia miltiorrhiza* is conducive to controlling the proteinuria level of IMN (Zheng et al., 2017). Compared to prednisone combined with cyclophosphamide in the treatment of adult IMN, Shenqi Granule has the same effect in controlling proteinuria (3.01 vs. 3.28 g/d), and the incidence of serious adverse events, such as pulmonary infection and liver injury, was significantly lower in the Shenqi Granule treatment group than the control group (Chen et al., 2013). Research on *Tripterygium wilfordii* and its active ingredients for IMN treatment is ongoing. A study found that the remission rates of *Tripterygium wilfordii* combined with low-dose prednisone after 3, 6, and 12 months were 74.4, 79.1, and 76.7%, respectively, which were significantly higher than the remission rates of *Tripterygium wilfordii* alone. *Tripterygium wilfordii* combined with low-dose prednisone is expected to become the first-line treatment scheme for IMN (Xu et al., 2015). IMN patients were treated with CNI + *Tripterygium* glycoside tablets (TWPs) and CNI + GCs. After 12 months of follow-up, there was no significant difference in the reduction in the rate of proteinuria or the positive rate of serum PLA2R between the groups. CNI combined with TWPs is another method for the treatment of IMN (Gao et al., 2021). In a study, 53 patients with IMN were treated with *Tripterygium wilfordii* polyglycosides (TWG) + prednisone and TAC + prednisone for 36 weeks. It was found that the partial remission rate, complete remission rate, and remission time of the groups were similar. Therefore, TWG combined with GCs is an effective treatment for IMN (Liu et al., 2015). In another study, 184 patients with IMN were treated with Mahuang Fuzi and Shenzhuo Decoction alone or with TCM combined with immunosuppressive therapy. After 3 years of follow-up, it was found that the remission rates of the groups were 65.5 and 59.6%, respectively (Dong et al., 2021).

For patients with refractory IMN, after 1 year of treatment with Jian Pi Qu Shi formula, the remission rate of nephropathy reached 80%, and the level of proteinuria decreased from 5.93 g/d to 1.99 g/d (Shi et al., 2018). In another study, 31 patients with refractory IMN were treated with Shulifenxiao for 12 months. It was found that the remission rate reached 90.9%, and the serum albumin and estimated glomerular filtration rate levels were significantly increased. After 2 years of follow-up, only two patients had experienced recurrence (Cui et al., 2021). Using Shen No. 9 recipe with Qingre Moshen Granules to treat IMN patients who did not respond to hormones and immunosuppressants can improve the cellular immune level and the abnormal expression of interleukin-2. After 24 weeks of treatment, the basic remission rate and total effective rate of 44 patients were 68.2 and 84.1%, respectively (Han et al., 2011).

### MCD

MCD accounts for 10%–15% of adult nephrotic syndrome cases. The recurrence rate of adult MCD is >50%, and 10%–20% of patients are resistant to steroids. In recent years, TCM has achieved certain curative effects in the adjuvant treatment of MCD (Wang et al., 2018). Yuebi Jiazhu Tang was used as adjuvant therapy for primary nephrotic syndrome in children. The remission rate and non-recurrence rate in the adjuvant treatment group with TCM were significantly higher than those in the non-treatment group (91.2 vs. 84.8% and 20.7 vs. 11.3%, respectively) (Wu et al., 2022). *Abelmoschus manihot* alone or combined with losartan potassium was used to treat patients with non-NS primary glomerular disease, including MCD and FSGS, and the results showed that *Abelmoschus manihot* could significantly reduce the level of proteinuria (Zhang et al., 2014). According to the syndrome differentiation of TCM, Wulin Powder, Zhenwu Decoction, and Zhibaidihuang Decoction were used to assist in the treatment of pediatric NS. The results showed that the levels of proteinuria and cholesterol in the integrated Chinese and Western medicine treatment group were significantly lower than those in the group treated with Western medicine alone (Wei, 1992). Yang et al. used Shenqi Dihuang Decoction to treat primary diseases, supplementing Qi and activating blood circulation to prevent thrombosis, supporting righteousness and dispelling pathogens to prevent infection, strengthening the spleen and stomach, improving malnutrition to treat MCD and related complications, and achieved obvious curative effects (Wu et al., 2020). There are few studies on adjuvant treatment of MCD with TCM or using TCM as the main means to treat MCD, with only small samples or case reports, and high-quality clinical studies with larger samples are needed to confirm in the future.

### FSGS

FSGS accounts for 40% of adult nephrotic syndrome cases. The treatment response is relatively poor, the incidence of hormone resistance is high, and long-term immunosuppressive treatment is prone to serious complications such as infection. The use of TCM in the treatment of primary FSGS has been studied in recent years. A case was treated with modified Fangji Huangqi Decoction for FSGS, and there was no recurrence after proteinuria was cured (Zhang and Yu, 2015). In one study, 70 children with primary nephrotic syndrome (including FSGS) were divided into a simple GC group (control group) and a GC combined with Liuwei Dihuang Pill Decoction group (TCM treatment group). The results showed that the decrease in proteinuria and the increase in serum albumin levels in the TCM treatment group were more obvious (3.75 g/d vs. 2.89 g/d and 37.9 g/L vs. 31.1 g/L, respectively). The total effective rates of the TCM group and the control group were 93.9 and 75.0%, respectively (*p* < 0.05). The recurrence rate in the control group was significantly higher than that in the control group (53.1 vs. 21.1%, *p* < 0.05) (Hou et al., 2021). Zhang et al. conducted a meta-analysis on the treatment of FSGS with the method of supplementing Qi and activating blood circulation. It was found that the treatment of supplementing Qi and activating blood circulation combined with Western medicine could significantly reduce the levels of proteinuria, serum creatinine, and urea nitrogen in patients with FSGS (*p* < 0.05) (Zhang et al., 2019). Unfortunately, most studies have used GC as the main treatment to observe whether TCM can enhance the efficacy or reduce recurrence, and the independent efficacy of TCM has not been observed. Clinical studies on treating podocytopathies with TCM are shown in Table 2.

**TABLE 2:** Clinical studies on treating podocytopathies with TCM.

Name	Compound TCM prescriptions	Main active ingredients	Clinical trial design	Podocytopathies	Remission rate	References
Shenqi particle	Radix Astragali, Radix Angelicae sinensis, Rhizoma Atractylodis, Rhizoma Atractylodis Macrocephalae, Radix Dioscoreae Oppositae, Sclerotium polypori Umbrellati, Sclerotium Poriae Cocos, *Bombyx* Batryticatus, Herba hedyotidis Diffusae, Semen coicis, Radix Codonopsitis Pilosulae, Radix Salvia miltiorrhizae, Hirudo Seu Whitmania	—	Intervention group: Shenqi particle; control group: prednisone and cyclophosphamide	IMN	—	Chen et al. (2013)
Jian Pi Qu Shi Formula	*Astragalus* membranaceus, Codonopsis pilosula, Poria cocos, Atractylodes macrocephala, Coix lacryma-jobi, Angelica sinensis, Polyporus umbellatus, Stephania tetrandra, *Dioscorea* nipponica Makino, Folium Perillae	—	JPQSF was administered orally twice a day for 6 months	Refractory patients with IMN	80%	Shi et al. (2018)
Jian Pi Qu Shi Formula	*Astragalus* membranaceus, Codonopsis pilosula, Poria cocos, Atractylodes macrocephala, Coix lacryma-jobi, Angelica sinensis, Polyporus umbellatus, Stephania tetrandra, *Dioscorea* nipponica Makino, Folium Perillae	—	JPQSF (TCM), immunosuppressant WM therapy	IMN	—	Lang et al. (2020)
Shen No. 9 Recipe (SR) combined with Qingre Moshen Granule (QMG)	—	—	SR (one dosage daily, oral administration in two portions) and QMG (each package each time, thrice daily) for 24 weeks	IMN patients with no efficacy after being treated with hormone or immunosuppressive agent	84.10%	Han et al. (2011)
Tripterygium wilfordii Hook F (TwHF)	—	Tripterygium wilfordii multi-glycosides	TWG + prednisone, TAC + prednisone, for 36 weeks	IMN	—	Liu et al. (2015)
Shulifenxiao Formula	Fructus forsythiae, Radix astragali, Bitter apricot, Heartleaf houttuynia, Magnolia officinalis, Rhizoma smilacis glabrae, Leech	—	Shulifenxiao treatment lasted 3–12 months	Refractory IMN	90.90%	Cui et al. (2021)
Mahuang Fuzi and Shenzhuo Decoction	Ephedra sinica Stapf, Aconitum carmichaeli Debx, boiled first, *Glycyrrhiza* uralensis Fisch, Zingiber officinale Rosc, Poria cocos, Atractylodes macrocephala Koldz	—	Mahuang Fuzi and Shenzhuo Decoction 1 dose daily, boiled with 400 ml water, and taken in the morning and evening for 6–36 months	IMN	59.60%	Dong et al. (2021)
Tripterygium wilfordii Hook	—	Tripterygium wilfordii polyglycoside	CNI + TWPs and CNI + GCs and followed up for more than 12 months	IMN	—	Gao et al. (2021)
Yuebi Jiazhu Tang; Ganlu Xiaodu Dan	Yuebi Jiazhu Tang: ephedra, gypsum, fresh ginger, licorice, largehead atractylodes, rhizome. Ganlu Xiaodu Dan: talc, Baical skullcap root, virgate wormwood herb, grass leaf sweet flag rhizome, akebia stem, blackberry lily rhizome, cardamon fruit, weeping forsythia capsule, tendril leaf, cablin patchouli herb, and peppermint	—	—	MCD	91.20%	Wu et al. (2022)
Abelmoschus manihot	—	Manihot	Manihot group, losartan group, combined group	MCD, FSGS	—	Zhang et al. (2014)
Shenqi Dihuang Decoction	*Astragalus* membranaceus, Codonopsis pilosula, cornel, dodder, Alisma orientalis, Poria cocos peel, Shiwei, Angelica sinensis, Salvia miltiorrhiza, and peach kernel	—	—	MCD	—	Yang et al. (2020)
Fangji Huangqi Decoction	Raw *Astragalus*, red beans, white gourd skin, Amomum villosum, lotus seed meat, Euryale ferox	—	—	FSGS	—	Zhang et al. (2015)
Supplementing Qi and activating blood circulation	—	—	—	FSGS	—	Zhang et al. (2019)

## Discussion

Patients with primary podocytosis are mostly characterized by massive proteinuria, high edema, hyperlipidemia, and hypoproteinemia. According to the recommendations of the Kidney Disease: Improving Global Outcomes (KDIGO) guidelines, the main treatment method at present is GC combined with immunosuppressive therapy. In recent years, the CD20 monoclonal antibody has also been widely used. However, in the treatment process of primary podocytosis, there is a risk of slow effects and poor curative effects, the treatment cycle is long, and recurrence rates are high. Long-term GC and immunosuppressive therapy are associated with a high risk of severe infection, liver damage, glucose and lipid metabolism disorders, and kidney damage, resulting in treatment failure of primary podocytosis and progression to ESRD.

For a long time, TCM has played an important role in the treatment of kidney disease. With the advancement of precision medicine, many active ingredients and functional mechanisms of TCMs have been studied, providing a scientific basis for the application of TCM. Unfortunately, due to the small sample size of many studies included in this review, and the lack of long-term follow-up and scientific design of the research results, the reliability of the research results is affected. Most studies on TCM in the treatment of podocytosis are still clinical observation studies, and most of the included studies do not have an in-depth study on the mechanism of action, the functional mechanisms of TCMs remain to be elucidated. Traditional Chinese medicine interventions are mostly compound preparations or decoction, and the dosage, form, and detailed medication information of the prescription are missing, resulting in poor repeatability of the research results. Most of the research subjects of TCM in the treatment of podocytosis are Chinese; in further research, we should verify the efficacy of TCM in other countries in the treatment of podocytosis, so as to eliminate the influence of race. Moreover, in addition to podocyte damage, there are other microenvironmental changes in primary podocyte disease. In the present study, we only reviewed podocyte injury treatment options from the perspective of TCM. In the future, attention should be paid to the functional mechanisms and efficacy of TCMs in the intervention of edema, hyperviscosity, immune regulation, and glucose and lipid metabolism disorders in patients with primary podocytosis. At the same time, fine design and international multi-center clinical research must be carried out to provide more evidence for the wide application of TCM in podocytosis.

## Glossary

TCM: traditional Chinese medicine
GBM: glomerular basement membrane
EMT: epithelial–mesenchymal transition
IMN: idiopathic membranous nephropathy
MCD: minimal change disease
FSGS: focal segmental glomerulosclerosis
CG: collapsing glomerulopathy
PMN: primary membranous nephropathy
NEP: neutral endopeptidase
PLA2R: M-type receptor for secreted phospholipase A2
THSD7A: thrombospondin type-1 domain-containing 7A
NELL-1: neural epidermal growth factor-like 1 protein
NCAM-1: neural cell adhesion molecule 1
CNTN1: contactin 1
IFN: interferon
NSAIDs: non-steroidal anti-inflammatory drugs
mTOR: mammalian target of rapamycin
CNIs: calcineurin inhibitors
CKD: chronic kidney disease
TUNEL: terminal deoxynucleotidyl transferase-mediated dUTP nick-end labeling
HIV: human immunodeficiency virus
APOL1: Apolipoprotein L1
CMV: Cytomegalovirus
EBV: Epstein–Barr virus
PI3K-AKT: phosphatidylinositol 3-kinase–RAC serine/threonine protein kinase
NF-κB: factor kappa-B
DKD: Diabetes kidney disease
DDP4: Dipeptidyl peptidase 4
TWPs: Tripterygium glycoside tablets
TWG: Tripterygium wilfordii polyglycosides
α-SMA: α-smooth muscle actin
TAC: tacrolimus.
